# Plasmin Inhibitors Prevent Leukocyte Accumulation and Remodeling Events in the Postischemic Microvasculature

**DOI:** 10.1371/journal.pone.0017229

**Published:** 2011-02-22

**Authors:** Christoph A. Reichel, Max Lerchenberger, Bernd Uhl, Markus Rehberg, Nina Berberich, Stefan Zahler, Matthias P. Wymann, Fritz Krombach

**Affiliations:** 1 Walter Brendel Centre of Experimental Medicine, Ludwig-Maximilians-Universität München, Munich, Germany; 2 Department of Pharmacy, Ludwig-Maximilians-Universität München, Munich, Germany; 3 Department of Biomedicine, Institute of Biochemistry and Genetics, University of Basel, Basel, Switzerland; Universität Würzburg, Germany

## Abstract

Clinical trials revealed beneficial effects of the broad-spectrum serine protease inhibitor aprotinin on the prevention of ischemia-reperfusion (I/R) injury. The underlying mechanisms remained largely unclear. Using *in vivo* microscopy on the cremaster muscle of male C57BL/6 mice, aprotinin as well as inhibitors of the serine protease plasmin including tranexamic acid and ε-aminocaproic acid were found to significantly diminish I/R-elicited intravascular firm adherence and (subsequent) transmigration of neutrophils. Remodeling of collagen IV within the postischemic perivenular basement membrane was almost completely abrogated in animals treated with plasmin inhibitors or aprotinin. In separate experiments, incubation with plasmin did not directly activate neutrophils. Extravascular, but not intravascular administration of plasmin caused a dose-dependent increase in numbers of firmly adherent and transmigrated neutrophils. Blockade of mast cell activation as well as inhibition of leukotriene synthesis or antagonism of the platelet-activating-factor receptor significantly reduced plasmin-dependent neutrophil responses. In conclusion, our data suggest that extravasated plasmin(ogen) mediates neutrophil recruitment *in vivo* via activation of perivascular mast cells and secondary generation of lipid mediators. Aprotinin as well as the plasmin inhibitors tranexamic acid and ε-aminocaproic acid interfere with this inflammatory cascade and effectively prevent postischemic neutrophil responses as well as remodeling events within the vessel wall.

## Introduction

Ischemia-reperfusion (I/R) injury is still the most common cause for organ dysfunction and failure after myocardial infarction, hemorrhagic shock, and transplantation. Neutrophil recruitment from the microvasculature to the perivascular tissue is a hallmark in the pathogenesis of I/R injury [1]. In this process, a variety of adhesion molecules, chemokines, and proteases have been implicated strictly controlling the single steps of leukocyte extravasation including rolling, firm adherence, and transendothelial migration [2], [3].

Plasmin is a serine protease which is released from the liver into the systemic circulation as the zymogen plasminogen. In addition to its well-known fibrinolytic properties, this protease has also been reported to play a critical role in various other physiological and pathophysiological processes including angiogenesis, wound healing, and inflammation. In this context, plasmin is suggested to initiate intracellular signaling pathways as well as to activate extracellular matrix (ECM) degrading enzymes ultimately facilitating cell adhesion and migration [4]–[6].

Despite recent concerns about the safety of the broad-spectrum serine protease inhibitor aprotinin [7]–[9], clinical trials revealed beneficial effects of this naturally occurring substance for the prevention of postischemic organ dysfunction [10]–[12]. Here, aprotinin has been suggested to suppress the transcription of genes which have been implicated in the evolution of the postischemic inflammatory response [13], [14]. The consequences for each single step of the leukocyte recruitment process during I/R, however, have not yet been studied.

Previous studies have implicated the serine protease plasmin as well as plasminogen activators in the regulation of leukocyte migration to the site of inflammation [4]–[6]. Interestingly, lysine analogues such as tranexamic acid or ε-aminocaproic acid have recently been reported to effectively and safely inhibit plasmin activity [7]–[9], [15]. The effect of these synthetic plasmin inhibitors on postischemic leukocyte responses has not yet been evaluated.

In the early reperfusion phase, remodeling processes within the perivenular basement membrane have been described which are thought to compromise microvascular integrity and to pave the way for the excessive leukocyte infiltration of reperfused tissue [16]. Due to its capability to disintegrate components of the venular basement membrane as well as to activate other ECM-degrading proteases, plasmin has been implicated in these events [6]. The effect of plasmin inhibitors and aprotinin on remodeling processes within the postischemic vessel wall has not yet been investigated.

Therefore, the objective of the present study was i) to systematically analyze the effect of the plasmin inhibitors tranexamic acid and ε-aminocaproic acid as well as of the broad-spectrum serine protease inhibitor aprotinin on each single step of the extravasation process of leukocytes as well as on remodeling events within the perivenular basement membrane during I/R and ii) to characterize the mechanisms underlying plasmin-dependent leukocyte responses *in vivo.*

## Results

### Effect of tranexamic acid, ε-aminocaproic acid, and aprotinin on postischemic leukocyte responses

**Figure 1:**
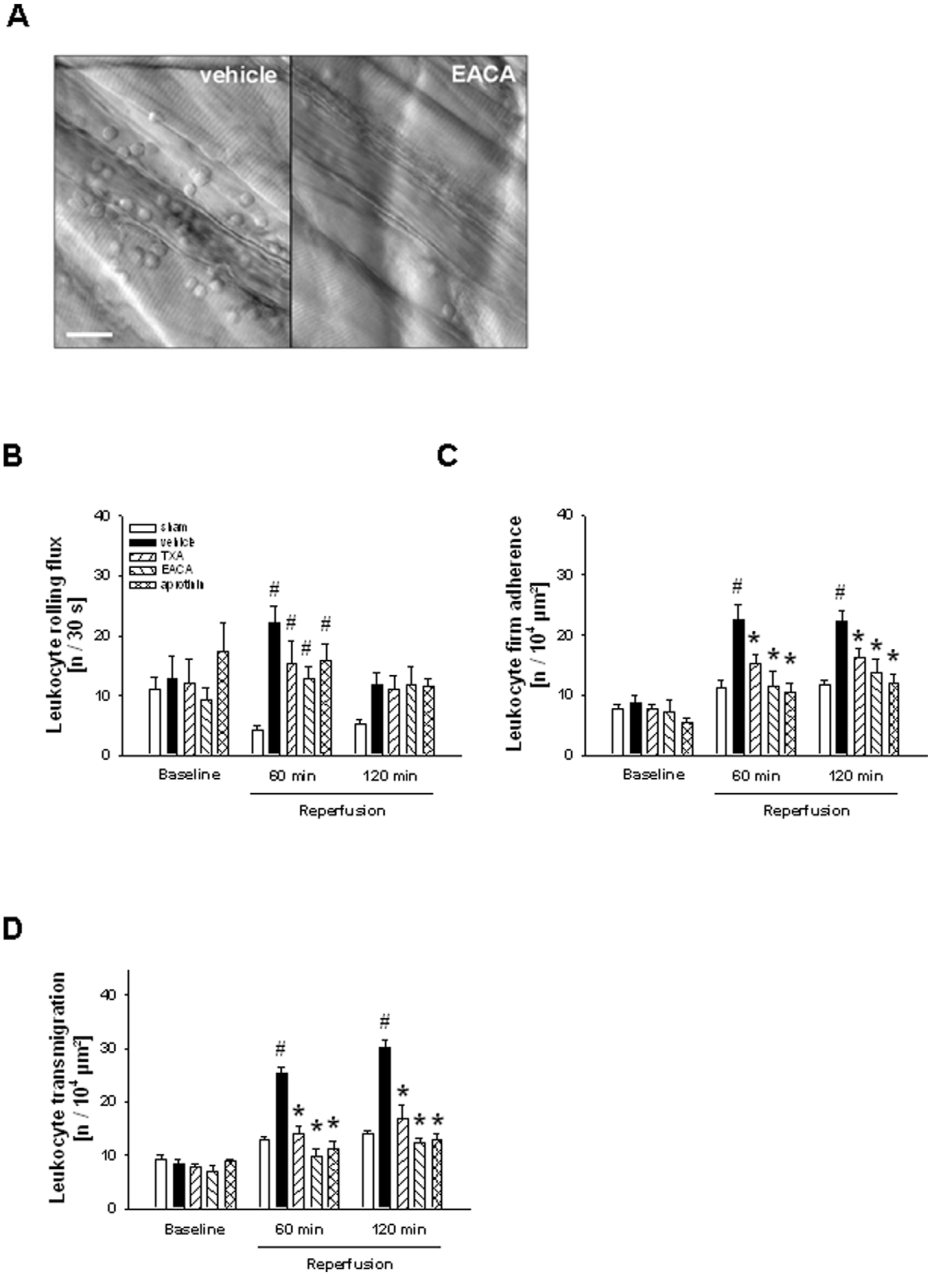
Effect of TXA, EACA, and aprotinin on postischemic leukocyte responses. Postcapillary venules in the postischemic cremaster muscle treated with ε-aminocaproic acid (EACA) or vehicle (A; scale bar 25 µm). Leukocyte rolling (B), firm adherence (C), and transmigration (D) in mice treated with tranexamic acid (TXA), EACA, or aprotinin undergoing I/R (mean±SEM; n = 6 per group; #p<0.05, vs. sham; *p<0.05, vs. vehicle).

By using near-infrared reflected-light oblique transillumination (RLOT) *in vivo* microscopy on the mouse cremaster muscle, effects of the plasmin inhibitors tranexamic acid and ε-aminocaproic acid as well as of the broad-spectrum serine protease inhibitor aprotinin on postischemic rolling, firm adherence, and transmigration of leukocytes were analyzed (**Fig. 1A**).

As is well known, surgical preparation of the cremaster muscle induced leukocyte rolling in postcapillary venules. After 30 min of ischemia and 60 min of reperfusion, there was a significant increase in numbers of rolling leukocytes as compared to sham-operated animals which returned to baseline values after 120 min of reperfusion. Treatment with tranexamic acid, ε-aminocaproic acid, or aprotinin did not significantly alter leukocyte rolling during the entire reperfusion phase (**Fig. 1B**).

Under baseline conditions prior to I/R, few leukocytes were found firmly adherent to the vessel wall of postcapillary venules (8.6±2.0/10^4^ µm^2^). In contrast, after 30 min of ischemia and 120 min of reperfusion, there was a significant elevation in numbers of firmly adherent leukocytes (22.6±2.6/10^4^ µm^2^) as compared to sham-operated controls (11.7±1.2/10^4^ µm^2^) This elevation was significantly reduced in animals treated with tranexamic acid (16.1±2.4/10^4^ µm^2^) or ε-aminocaproic acid (13.8±3.1/10^4^ µm^2^) and almost completely abolished in aprotinin-treated (11.9±2.2/10^4^ µm^2^) animals (**Fig. 1C**).

Prior to I/R, only few transmigrated leukocytes were detected within the perivascular tissue (8.3±1.3/10^4^ µm^2^). In response to I/R (30/120 min), the number of transmigrated leukocytes was significantly increased (30.1±2.3/10^4^ µm^2^) as compared to sham-operated controls (14.0±0.7/10^4^ µm^2^). Similar to our results for leukocyte firm adherence, the postischemic increase in leukocyte transmigration was significantly attenuated in mice treated with tranexamic acid (17.0±3.4/10^4^ µm^2^) and completely abrogated in ε-aminocaproic acid- (12.2±1.5/10^4^ µm^2^) or aprotinin-treated mice (12.8±2.0/10^4^ µm^2^). Similar results for leukocyte firm adherence and transmigration were found already after 60 min of reperfusion (**Fig. 1D**).

### Effect of tranexamic acid, ε-aminocaproic acid, and aprotinin on postischemic remodeling of the perivenular basement membrane

**Figure 2:**
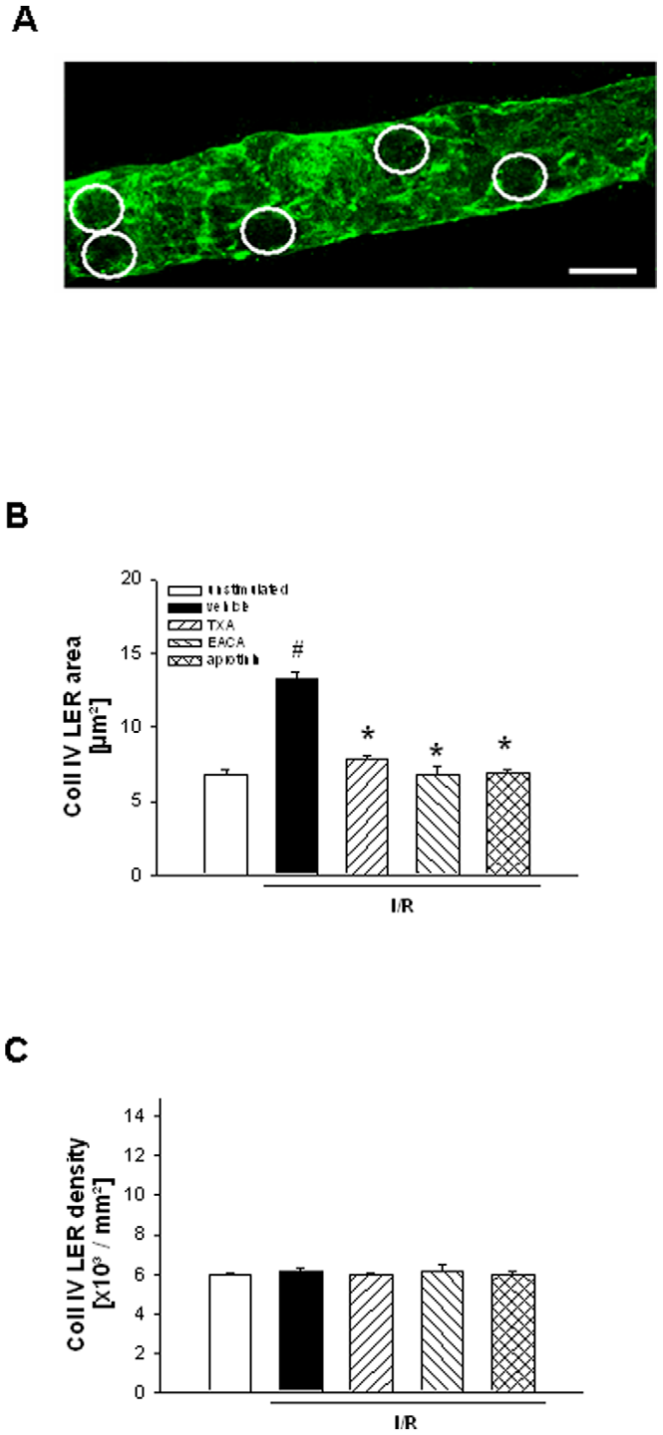
Effect of TXA, EACA, and aprotinin on postischemic remodeling of the perivenular basement membrane. Postcapillary venule in the postischemic cremaster muscle immunostained for collagen IV. White rings show low-expression regions (LER; A; scale bar 10 µm). Size (B) and density (C) of LER in mice treated with TXA, EACA, or aprotinin undergoing I/R (mean±SEM; *n* = 4 per group; #*p*<0.05, vs. unstimulated; *p<0.05, vs. vehicle).

To characterize the expression profile of collagen IV within the perivascular basement membrane, immunofluorescence staining and confocal laser scanning microscopy were performed in tissue samples of the cremaster muscle. In unstimulated animals, a discontinuous expression of collagen IV was detected in postcapillary venules (**Fig. 2A**). Analysis of intensity profiles demonstrated regions of low fluorescence intensity (less than 60% of average fluorescence intensity/unit area of the entire vessel segment). These low-expression regions (LER) were detected at a density of 6.0±0.1×10^3^/mm^2^ (**Fig. 2C**) and had an average size of 6.8±0.4 µm^2^ (**Fig. 2B**). Interestingly, I/R did not alter the average density of these collagen IV LER, but caused a significant enlargement of the average site size of collagen IV LER (13.3±0.4 µm^2^). This enlargement was almost completely abolished in mice treated with tranexamic acid (7.9±0.2 µm^2^), ε-aminocaproic acid (6.8±0.6 µm^2^), or aprotinin (6.9±0.2 µm^2^).

### Effect of tranexamic acid, ε-aminocaproic acid, and aprotinin on postischemic microvascular leakage in the initial reperfusion phase

**Figure 3:**
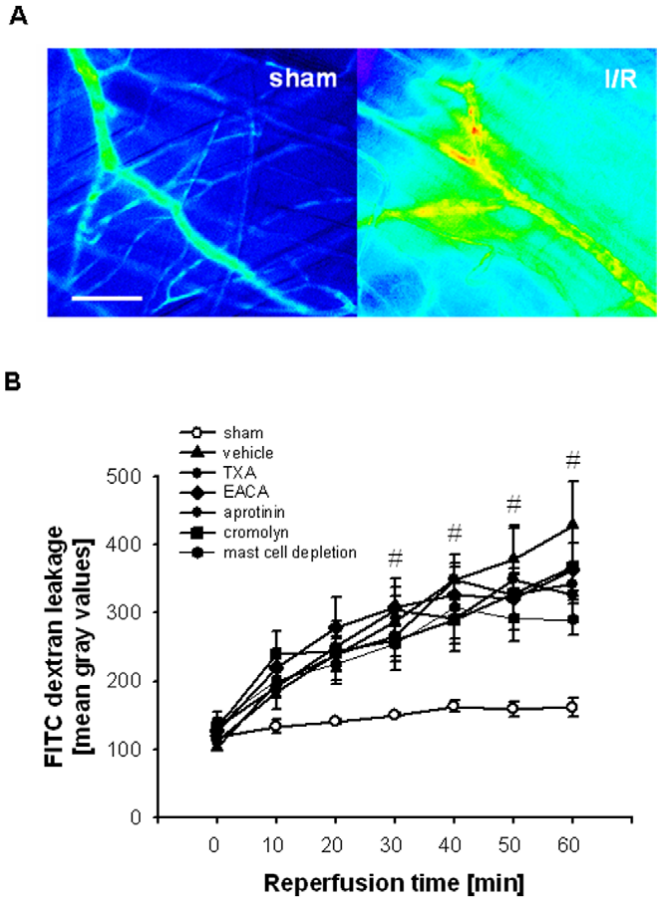
Effect of TXA, EACA, and aprotinin on postischemic microvascular leakage. FITC dextran leakage in the postischemic cremaster muscle (A; scale bar 100 µm). FITC dextran leakage in mice treated with TXA, EACA, aprotinin, or cromolyn as well as in mast cell-depleted mice undergoing I/R (B; mean±SEM for *n* = 4 per group; #p<0.05, vs. sham).

As a measure of microvascular permeability, leakage of FITC dextran to the postischemic cremaster muscle was analyzed (**Fig. 3A**). In the initial reperfusion phase, there was a time-dependent, significant increase in the leakage of FITC dextran as compared to sham-operated controls. This increase did not significantly differ from that observed in mice treated with the plasmin inhibitors tranexamic acid or ε-aminocaproic acid, with the broad-spectrum serine protease inhibitor aprotinin, with an inhibitor of mast cell degranulation (cromolyn), or in mast cells-depleted mice (**Fig. 3B**).

### Mast cell activation in response to I/R

**Figure 4:**
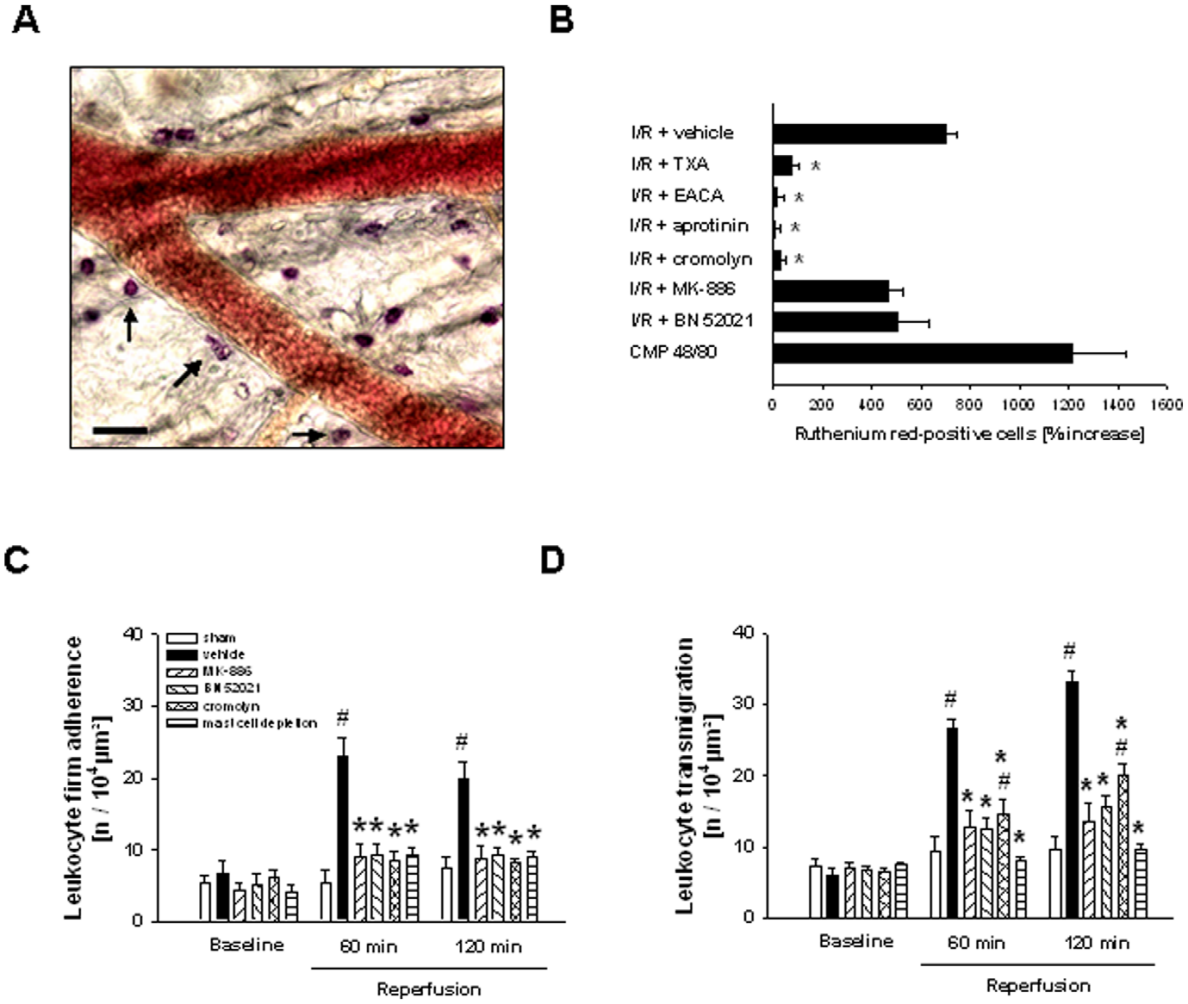
Role of mast cells and lipid mediators for postischemic leukocyte responses. Cremasteric ruthenium-red-positive mast cells (A; arrows). The number of ruthenium red-positive cells in mice treated with TXA, EACA, aprotinin, cromolyn, MK-886, or BN52021 undergoing I/R (B). Leukocyte firm adherence (C) and transmigration (D) in mast cell-depleted mice as well as in mice treated with cromolyn, BN52021, or MK-886 undergoing I/R (mean±SEM for *n* = 4–5 per group; #p<0.05, vs. sham; *p<0.05, vs. vehicle).

As a measure of mast cell activation *in vivo*, ruthenium red staining of the cremaster muscle was performed (**Fig. 4A**). In response to I/R (30/120 min), there was a significant elevation (702.3±43.3%) in the number of ruthenium red-positive cells as compared to sham-operated animals. This elevation was almost completely abolished in animals treated with an inhibitor of mast cell degranulation (cromolyn; 31.7±20.2%) as well as with the plasmin inhibitors tranexamic acid (75.0±30.5%) and ε-aminocaproic acid (10.2±31.1%), or with the serine protease inhibitor aprotinin (6.9±18.6%). Blockade of the PAF receptor (BN52021; 469.3±54.1%) or inhibition of leukotriene synthesis (MK-886; 506.8±126.7%) only partially reduced the number of cremasteric ruthenium red-positive cells (**Fig. 4B**). As a positive control, treatment with compound 48/80 (CMP 48/80) strongly enhanced the number of ruthenium red-positive mast cells (1214.8±219.8%) as compared to unstimulated controls. Conversely, in mast cell-depleted mice only few single ruthenium red-positive mast cells were detected (data no shown).

### Role of mast cells, leukotrienes, and platelet-activating factor on postischemic leukocyte responses

In further experiments, the effect of mast cell depletion, of an inhibitor of mast cell degranulation (cromolyn), of an inhibitor of leukotriene synthesis (MK-886), and of a PAF receptor antagonist (BN52021) on postischemic rolling, firm adherence, and transmigration of leukocytes was analyzed. Whilst mast cell depletion or treatment with cromolyn, MK-886, or BN52021 did not significantly alter the number of leukocytes rolling in postcapillary venules of the postischemic cremaster muscle (data not shown), the I/R (30/120 min)-elicited elevation in numbers of firmly adherent (**Fig. 4C**) and (subsequently) transmigrated (**Fig. 4D**) leukocytes was significantly reduced. Similar results for leukocyte firm adherence and transmigration were obtained already after 60 min of reperfusion.

### Effect of plasmin on surface expression of CD11b and CD62L on neutrophils

**Figure 5:**
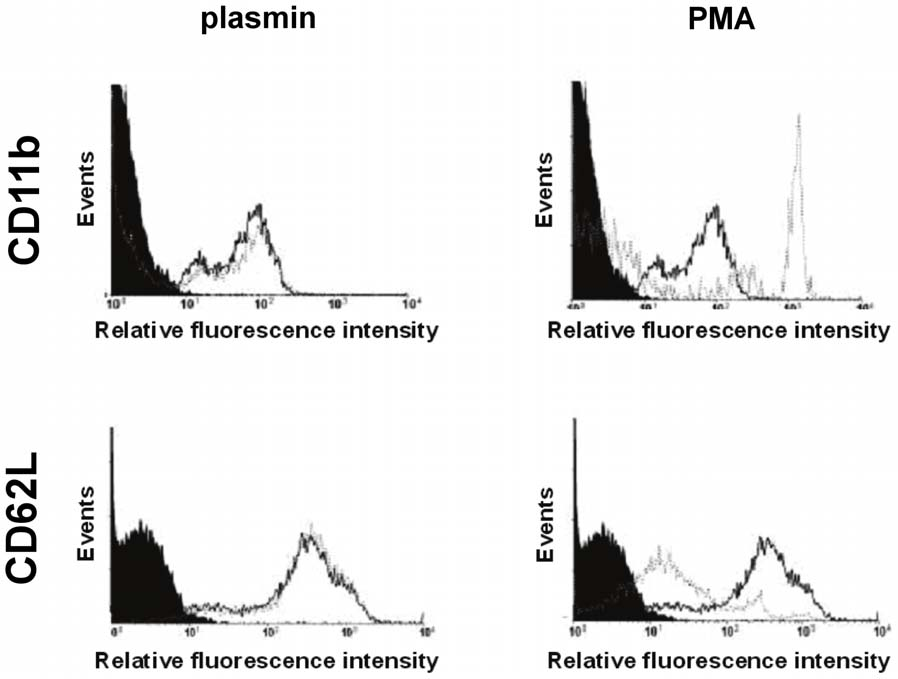
Effect of plasmin on neutrophil activation. Fluorescence histograms for expression of CD11b/Mac-1 and CD62L/L-selectin on murine neutrophils undergoing stimulation with plasmin, PMA (open histograms, broken lines), or vehicle (open histograms, solid lines) as compared to control IgG (solid histograms).

The effect of plasmin on the expression of adhesion molecules CD11b/Mac-1 and CD62L/L-selectin on murine neutrophils was analyzed by flow cytometry (**Fig. 5**). Co-incubation with plasmin (for all concentrations analyzed) did not significantly alter the expression of CD11b/Mac-1 and CD62L/L-selectin on the surface of murine neutrophils isolated from the peripheral blood as compared to unstimulated controls. In contrast, the potent neutrophil stimulant PMA was able to significantly enhance cell surface expression of CD11b/Mac-1 and shedding of CD62L/L-selectin.

### Effect of intravascular vs. extravascular application of plasmin on leukocyte rolling, firm adherence, and transmigration

**Figure 6:**
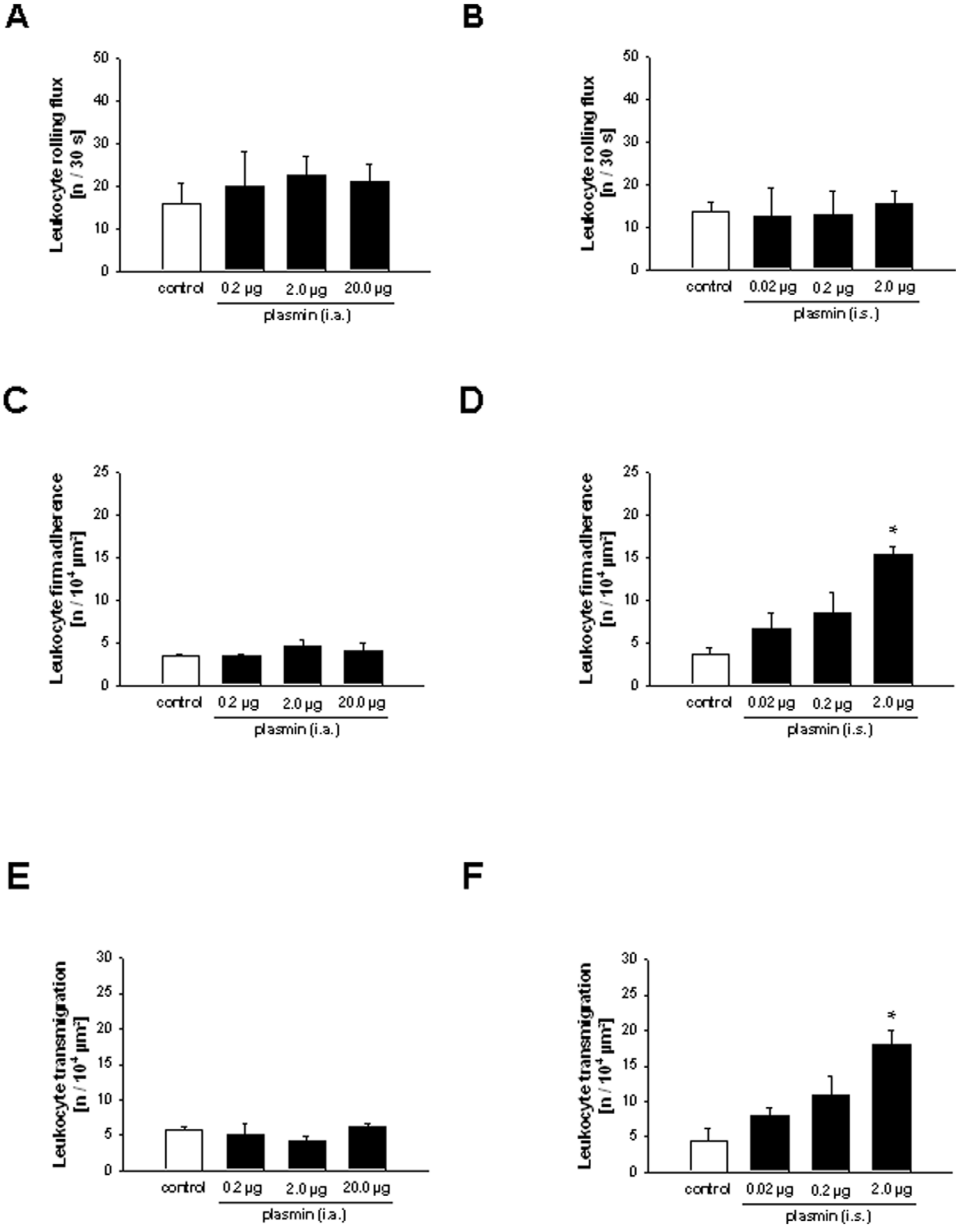
Effect of plasmin on leukocyte rolling, firm adherence, and transmigration. Leukocyte responses in the cremaster muscle after intraarterial (A, C, E) or intrascrotal (B, D, F) injection of plasmin (mean±SEM for *n* = 3 per group; *p<0.05, vs. control).

To further investigate the mode of action of plasmin *in vivo*, murine plasmin was applied either to the vascular compartment (intraarterial injection) or to the extravascular compartment (intrascrotal injection). Intravascular administration of plasmin did not alter rolling, firm adherence, and transmigration of leukocytes as compared to controls (at all concentrations investigated; **Fig. 6A, C, E**). Interestingly, extravascularly applied plasmin did not alter leukocyte rolling, but induced a dose-dependent increase in numbers of firmly adherent and transmigrated leukocytes as compared to vehicle-treated controls (**Fig. 6B, D, F**). Since a dose of 2.0 µg was able to elicit a robust elevation in firm adherence and (subsequent) transmigration of leukocytes, this concentration was used in further experiments.

### Mast cell activation in response to plasmin

**Figure 7:**
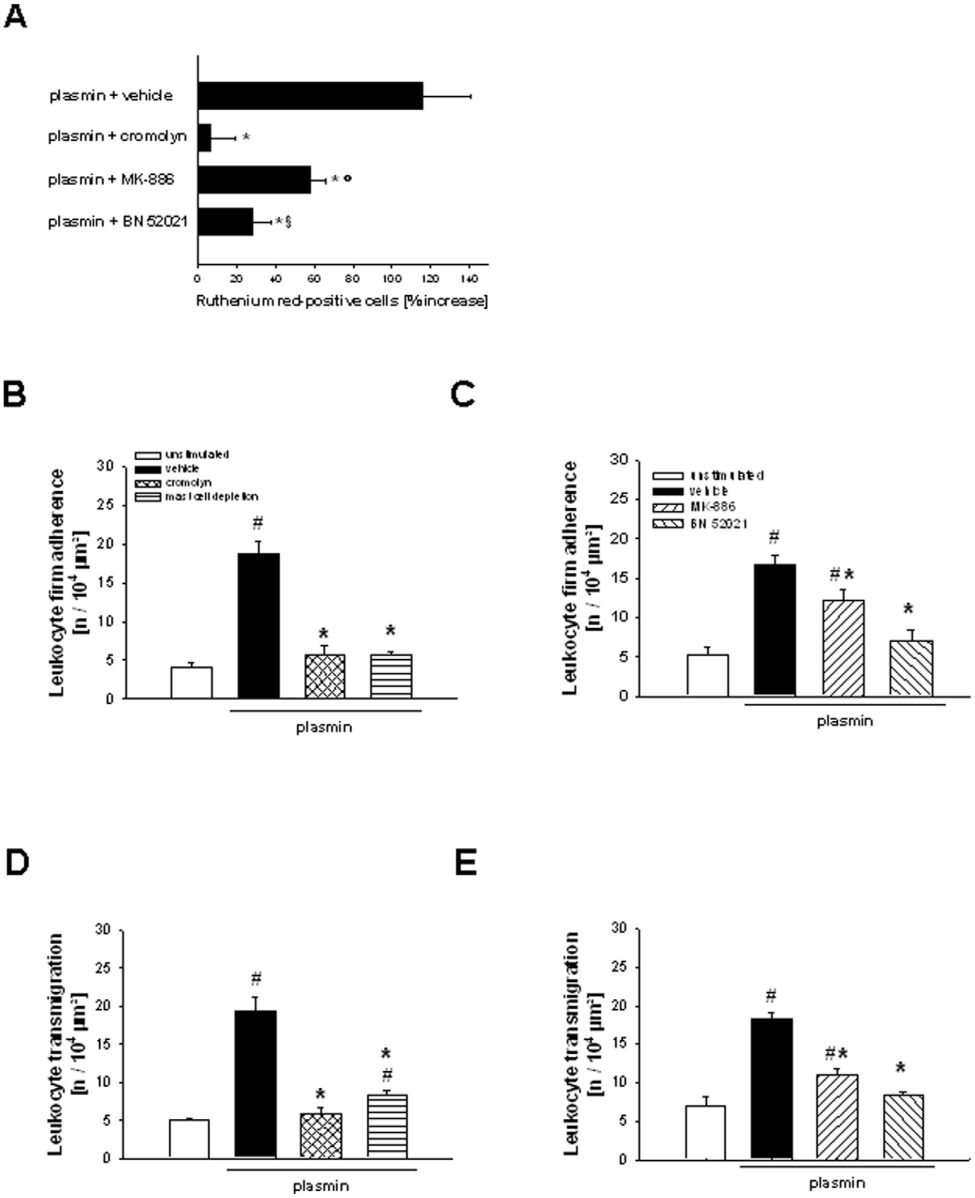
Role of mast cells and lipid mediators for plasmin-dependent leukocyte responses. The number of ruthenium red-positive cells in the cremaster muscle of mice treated with cromolyn, MK-886, or BN52021 undergoing stimulation with plasmin (A). Leukocyte responses in the cremaster muscle of mast cell-depleted mice as well as of mice treated with cromolyn (B, D), MK-886, BN52021 (C, E), or vehicle undergoing stimulation with plasmin (mean±SEM for *n* = 4–6 per group; #p<0.05, vs. unstimulated; *p<0.05, vs. vehicle; §p<0.05, vs. MK-886; °p<0.05, vs. cromolyn).

In separate experiments, mast cell activation was analyzed upon stimulation with plasmin. Four hours after intrascrotal injection of plasmin, the number of cremasteric ruthenium red-positive cells was significantly increased (115.9±24.9%) as compared to unstimulated controls (**Fig. 7A**). This increase was almost completely abolished in cromolyn-treated animals (6.5±13.0%), but only partially reduced in animals treated with MK-886 (57.6±8.1%) or BN52021 (28.5±8.9%). In mast cell-depleted animals, only few single ruthenium red-positive cells were detected (data not shown).

### Effect of plasmin on tissue expression of 5-lipoxygenase and lyso-PAF-acetyltransferase

Using RT-PCR, the effect of plasmin on RNA expression of 5-lipoxygenase (5-LO) and lyso-PAF-acetyltransferase (LPCAT), enzymes facilitating the synthesis of leukotrienes and PAF, respectively, was analyzed in the mouse cremaster muscle. In response to plasmin, RNA expression of 5-LO (331.8±23.1%) and LPCAT (484.3±68.4%) was strongly enhanced as compared to unstimulated controls (data not shown).

### Role of mast cells for plasmin-elicited leukocyte responses

Using near-infrared RLOT *in vivo* microscopy on the cremaster muscle, the effect of mast cell deficiency or treatment with the mast cell stabilizer cromolyn on plasmin-elicited leukocyte responses was analyzed. Four hours after intrascrotal injection of plasmin, no significant differences were observed in numbers of rolling leukocytes among all experimental groups (data not shown). In contrast, the numbers of firmly adherent (13.7±1.7/10^4^ µm^2^) and (subsequently) transmigrated leukocytes (16.3±2.2/10^4^ µm^2^) were found to be significantly increased upon stimulation with plasmin as compared to unstimulated controls (5.3±0.9/10^4^ µm^2^; 6.3±1.3/10^4^ µm^2^). This increase was almost completely abolished in animals treated with cromolyn (4.6±1.0/10^4^ µm^2^; 7.3±0.7/10^4^ µm^2^) or in mast cell-depleted animals (5.8±0.4/10^4^ µm^2^; 8.4±0.6/10^4^ µm^2^; **Fig. 7B, D**).

### Role of leukotrienes and platelet-activating-factor for plasmin-elicited leukocyte responses

To characterize the role of secondarily generated lipid mediators in plasmin-elicited leukocyte responses, the PAF receptor antagonist BN52021 as well as the inhibitor of leukotriene synthesis MK-886 was used. No significant differences were detected among all experimental groups in numbers of rolling leukocytes (data not shown). In contrast, four hours after intrascrotal injection of plasmin, there was a significant increase in the number of firmly adherent leukocytes (17.0±1.2/10^4^ µm^2^) and transmigrated leukocytes (18.4±0.8/10^4^ µm^2^) as compared to unstimulated controls (5.3±0.9/10^4^ µm^2^; 6.9±1.3/10^4^ µm^2^). This increase was found to be significantly diminished in animals treated with MK-886 (12.1±1.4/10^4^ µm^2^; 11.0±0.9/10^4^ µm^2^) or BN52021 (7.1±1.4/10^4^ µm^2^; 8.4±0.4/10^4^ µm^2^; **Fig. 7C, E**).

### Phenotyping of transmigrated leukocytes

To identify the phenotype of transmigrated leukocytes, immunostaining for CD45 (common leukocyte antigen), Ly-6G (neutrophils), and F4/80 (monocytes/macrophages) of cremasteric tissue samples was performed. In response to I/R as well as upon intrascrotal injection of plasmin, over 80% of transmigrated leukocytes were positive for Ly-6G and about 20% of transmigrated leukocytes were positive for F4/80, respectively (**Tab. 1**).

**Table 1:** Phenotyping of transmigrated leukocytes was performed by immunostaining for CD45 (common leukocyte antigen), Ly-6G (neutrophils), and F4/80 (monocytes/macrophages) of cremasteric tissue samples as detailed in *Material and Methods* (mean±SEM for *n* = 6 per group).

	Ly-6G^+^ cells(% of CD45^+^ cells)	F4/80^+^ cells(% of CD45^+^ cells)
**I/R**	83.5±0.1	18.3±0.1
**Plasmin**	83.3±0.1	22.5±0.1

### Microhemodynamic parameters and systemic leukocyte counts

To assure intergroup comparability, quantification of inner vessel diameters, blood flow velocities, and wall shear rates of analyzed postcapillary venules as well as systemic leukocyte counts was performed (**Tab. 2**). No significant differences were detected among all experimental groups.

**Table 2:** Systemic leukocyte counts as well as microhemodynamic parameters, including inner vessel diameter, blood flow velocity, and wall shear rate were obtained as detailed in *Material and Methods* (mean±SEM for *n* = 3–6 per group).

		Inner diameter [µm]	Vmean [mm/s]	Wall shear rate [s-1]	Systemic leukocyte counts [x 10^6^ µl^−1^]
**I/R**	**sham**	25.6±0.3	1.2±0.1	1782.7±136.7	2.2±0.4
	**vehicle**	25.9±0.2	1.2±0.1	1978.9±166.3	2.5±0.5
	**TXA**	25.9±0.4	1.3±0.2	1875.0±50.7	3.2±0.7
	**EACA**	26.0±0.5	1.4±0.1	1740.8±69.3	2.9±0.4
	**aprotinin**	25.8±0.3	1.3±0.1	1961.8±90.3	3.4±0.7
**I/R**	**sham**	25.9±1.1	1.3±0.1	2011.6±71.3	3.2±1.1
	**vehicle**	26.1±0.4	1.3±0.1	2011.1±82.0	3.1±0.6
	**MK-886**	25.0±0.8	1.2±0.1	1923.8±214.3	3.0±0.7
	**BN 52021**	24.0±1.0	1.1±0.1	1903.6±397.4	3.3±1.5
	**cromolyn**	24.2±0.6	1.1±0.1	1827.2±137.0	3.5±0.4
	**mast cell depletion**	24.2±0.7	1.3±0.1	2165.8±43.0	3.6±0.7
**plasmini.a.**	**control**	26.1±0.4	1.5±0.1	2217.1±231.3	4.6±1.1
	**0.2 µg**	26.0±0.1	1.2±0.1	1856.0±69.1	4.8±1.0
	**2.0 µg**	25.6±0.2	1.4±0.1	2103.8±106.2	3.8±0.8
	**20.0 µg**	26.1±0.4	1.4±0.1	2124.4±39.5	4.9±0.6
**plasmini.s.**	**control**	26.0±1.5	1.5±0.1	1689.5±122.8	2.9±0.9
	**0.02 µg**	25.9±0.4	1.2±0.1	1766.1±183.4	5.0±1.3
	**0.2 µg**	26.5±0.4	1.3±0.1	1879.1±55.2	5.8±0.8
	**2.0 µg**	25.2±0.4	1.1±0.1	1740.3±160.4	7.7±2.0
**plasmini.s.**	**control**	26.2±0.8	1.3±0.1	1972.8±150.3	4.8±1.1
	**vehicle**	26.0±0.4	1.3±0.1	1929.9±53.4	5.4±1.3
	**BN 52021**	26.0±0.2	1.3±0.1	2023.3±115.7	5.9±0.8
	**MK-886**	26.0±0.4	1.3±0.1	1995.9±83.5	3.1±0.5
	**cromolyn**	26.4±0.5	1.3±0.1	1920.9±197.8	5.1±0.9
	**mast cell depletion**	25.2±0.6	1.3±0.1	2047.1±69.1	3.6±0.6

### Expression of the plasmin(ogen) receptor histone protein H2B on BMMCs

Using flow cytometry as well as immunostaining and confocal laser scanning microscopy, expression of the major plasmin(ogen) receptor *histone protein H2B* was detected on the surface of bone marrow-derived mast cells (BMMCs; data not shown).

## Discussion

Restoration of blood flow is the overall goal for successful organ transplantation as well as for the treatment of myocardial infarction, hemorrhagic shock, and stroke. As a consequence of this inevitable approach, however, neutrophils accumulate within the postischemic microvasculature and compromise reperfusion of the affected organ. Subsequently, transmigrating neutrophils release reactive oxygen species, cytokines, and proteases, impairing microvascular integrity and promoting postischemic tissue injury [1]. Notably, extravasated neutrophils also contribute to tissue healing and regeneration [17] collectively emphasizing neutrophil recruitment as a key event in the pathogenesis of I/R injury.

Using different animal models, the serine protease plasmin as well as plasmin activators have been implicated particularly in the migration of monocytes, but also in the recruitment of neutrophils [4]–[6]. Moreover, clinical trials revealed beneficial effects of the broad-spectrum serine protease inhibitor aprotinin for the prevention of postischemic organ dysfunction after coronary revascularization [10]–[12]. In this context, aprotinin has been reported to suppress the transcription of genes which are supposed to play a major role in the postischemic inflammatory response [13], [14]. The resulting consequences for each single step of the leukocyte recruitment process, however, remained unclear.

Using near-infrared RLOT *in vivo* microscopy on the mouse cremaster muscle, we systematically analyzed the effects on postischemic rolling, firm adherence, and transmigration of leukocytes of the broad-spectrum serine protease inhibitor aprotinin, a naturally occurring bovine protein, as well as of the synthetic plasmin inhibitors tranexamic acid and ε-aminocaproic acid. Our experimental data demonstrate that aprotinin as well as the plasmin inhibitors do not significantly alter leukocyte rolling in the early reperfusion phase. In contrast, firm adherence and (subsequent) transmigration of neutrophils to the postischemic tissue was found to be significantly diminished in animals treated with tranexamic acid, ε-aminocaproic acid, or aprotinin. These findings are in agreement with previous observations as elevated myeloperoxidase levels in the postischemic myocardium were significantly reduced upon treatment with aprotinin [13], [14]. It is interesting that aprotinin as well as the plasmin inhibitors suppressed postischemic neutrophil recruitment already on the level of intravascular adherence whilst under different inflammatory conditions (e.g. stimulation with fMLP, LTB_4_, or IL-1β) aprotinin has been reported to selectively diminish transendothelial migration of neutrophils [18]–[20]. Consequently, these data point to a stimulus-specific effect of aprotinin on the single steps of the extravasation process of neutrophils.

Recently, remodeling processes within the postischemic vessel wall have been described which are thought to be critically involved in the pathogenesis of I/R injury [16]. Specifically, there are regions within the basement membrane of postcapillary venules where the expression of collagen IV, a main structural component of venular basement membranes, is significantly lower than the average vascular level [16], [18]. In response to I/R, these low-expression regions (LERs) of collagen IV become strongly enlarged thereby compromising microvascular integrity as well as promoting the excessive leukocyte infiltration of reperfused tissue. Interestingly enough, treatment with tranexamic acid, ε-aminocaproic acid, or aprotinin almost completely abolished these postischemic remodeling events within the perivenular basement membrane and might thereby significantly contribute to the prevention of I/R injury. Whether these effects of the plasmin inhibitors are the result of a direct inhibition of plasmin-mediated degradation of collagen IV (e.g. by inhibiting plasmin activity on the surface of transmigrating neutrophils or in the extravasating plasma) or the consequence of diminished firm adherence and (subsequent) transmigration of neutrophils cannot clearly be answered in this *in vivo* study. Collectively, our experimental data demonstrate that the plasmin inhibitors tranexamic acid and ε-aminocaproic acid as well as the broad-spectrum serine protease inhibitor aprotinin effectively prevent intravascular firm adherence as well as (subsequent) transmigration of neutrophils to the reperfused tissue and protect the microvasculature from postischemic remodeling events. Notably, treatment with aprotinin has recently been reported to be associated with transient renal failure and other complications in critically ill patients [7]–[9]. In consideration of the comparatively mild side effects, the strong anti-inflammatory potency, and the considerably low costs of the lysine analogues tranexamic acid and ε-aminocaproic acid, the use of these drugs might be favored for the prevention of I/R injury.

Although the effects of aprotinin and the plasmin inhibitors on postischemic neutrophil responses as well as on remodeling events within the vessel wall have now been elucidated, the mechanisms underlying plasmin-dependent neutrophil recruitment *in vivo* remain poorly understood. Plasmin(ogen) is primarily generated in the liver and subsequently released into the systemic circulation where it is known to play a major role in the fibrinolytic system [4]–[6]. Our *in vivo* data demonstrate that intravascularly circulating plasmin is not able to induce significant leukocyte responses. In this context, physiological plasmin antagonists such as α_2_-antiplasmin are thought to minimize excessive proteolytic activity of plasmin within the vascular compartment and might thereby prevent inflammatory effects of this protease under physiological conditions. In the initial reperfusion phase, however, permeability of the postischemic microvasculature rapidly increases enabling plasmin(ogen) to extravasate to the perivascular tissue. Interestingly, extravascular administration of plasmin caused a dose-dependent elevation in numbers of firmly adherent and transmigrated neutrophils. Our results confirm previous observations as intrastriatal injection of plasmin has been reported to induce neutrophil infiltration of the brain [21]. Consequently, these data indicate that intravascularly circulating plasmin(ogen) does not exert inflammatory effects until it extravasates to the perivascular tissue. Moreover, we found that incubation with plasmin did not alter surface expression of CD11b/Mac-1 and CD62L/L-selectin on murine neutrophils suggesting that plasmin is not able to directly activate neutrophils. Notably, it cannot be excluded that I/R creates a favorable environment for direct actions of plasmin on neutrophils and it might also be possible that plasmin is able to induce affinity changes of integrins ultimately facilitating extravasation of neutrophils. In addition, it might be possible that receptor-bound plasminogen presented on the surface of circulating leukocytes might already be activated within the vascular compartment during I/R and might thereby contribute to leukocyte extravasation as hypothetized by previous *in vitro* studies [5], [6].

Because of their close vicinity to the vascular endothelium and their ability to generate an abundance of inflammatory mediators (e.g. leukotrienes and PAF), tissue mast cells are considered as key players in the postischemic inflammatory response [22], [23]. In this context, it is worth to be noted that the involvement of mast cells might be variable in different organs since tissue specific diversity in the phenotype, density, and distribution of mast cells has previously been reported [24]. In our experiments, we found that treatment with aprotinin as well as with the plasmin inhibitors almost completely prevents postischemic activation of mast cells. Furthermore, we demonstrate that plasmin is able to activate perivascular mast cells *in vivo* extending previous observations as plasmin has been reported to directly activate cultured mast cells [25]. In line with these results, we also show that blockade of mast cell activation almost completely abolished plasmin-dependent intravascular firm adherence and (subsequent) transmigration of neutrophils. Moreover, it is interesting that treatment with aprotinin or with the plasmin inhibitors as well as blockade of mast cell activation did not affect microvascular leakage in the early reperfusion phase. Accordingly, interaction of extravasated plasminogen with plasminogen receptors (e.g. *histone protein H2B* [26]) on perivascular mast cells is suggested to accelerate the conversion of plasminogen to plasmin, to protect plasmin from inactivation by endogenous inhibitors, and to enhance the biological activity of this protease [5], [6]. Collectively, these data indicate a divergent role of plasmin in the regulation of postischemic leukocyte recruitment and microvascular permeability and, moreover, strongly suggest that extravasated plasmin(ogen) mediates neutrophil recruitment *in vivo* indirectly via activation of perivascular mast cells.

Following recent *in vitro* studies, surface-bound plasmin is supposed to specifically interact with different cell-surface receptors (e.g. protease-activated receptors, integrins, annexin A2) [27], [28], to activate intracellular signaling pathways [29], and to induce the generation of inflammatory mediators [30], [31]. Here, we demonstrate that plasmin is able to induce the expression of *5-lipoxygenase* and *lyso-PAF-acetyltransferase*, key enzymes controlling the synthesis of leukotrienes and PAF, respectively. Moreover, inhibition of leukotriene synthesis or blockade of the PAF receptor significantly diminished plasmin-dependent firm adherence and (subsequent) transmigration of neutrophils. Thus, our results indicate that plasmin facilitates neutrophil extravasation *in vivo* via endogenous generation of lipid mediators. Consequently, in the early reperfusion phase, extravasated plasmin(ogen) is suggested to induce the generation of leukotrienes and PAF which, in turn, directly activate neutrophils [32] and promote intravascular adherence as well as (subsequent) transmigration of these inflammatory cells in postischemic tissue. Since inhibition of leukotriene synthesis or blockade of the PAF receptor only partially reduced plasmin- as well as I/R-elicited activation of mast cells, the postischemic generation of lipid mediators is, at least in part, suggested to occur downstream of mast cell activation.

In conclusion, our experimental data suggest that extravasated plasmin(ogen) mediates firm adherence and (subsequent) transmigration of neutrophils to the reperfused tissue indirectly through activation of perivascular mast cells and a sequential generation of leukotrienes and PAF. The plasmin inhibitors tranexamic acid and ε-aminocaproic acid as well as the broad-spectrum serine protease inhibitor aprotinin are thought to interfere with this inflammatory cascade and effectively prevent intravascular accumulation and (subsequent) transmigration of neutrophils to the reperfused tissue as well as protect the microvasculature from postischemic remodeling events. These findings provide novel insights into the mechanisms underlying the postischemic inflammatory response and highlight the use of plasmin inhibitors as a potential therapeutic approach for the prevention of I/R injury.

## Materials and Methods

### Animals

Male C57BL/6 mice were purchased from Charles River (Sulzfeld, Germany). All experiments were performed with male mice at the age of 10–12 weeks. Animals were housed under conventional conditions with free access to food and water. All experiments were performed according to German legislation for the protection of animals (approved by the government of Upper Bavaria; permit number 55.2-1-54-2531-84/09).

### Surgical procedure

The surgical preparation was performed as originally described by Baez with minor modifications [33], [34]. Mice were anesthetized using a ketamine/xylazine mixture (100 mg/kg ketamine and 10 mg/kg xylazine), administrated by i.p. injection. The right carotid and the left femoral artery were cannulated in a retrograde manner for continuous blood pressure monitoring and for administration of microspheres and drugs (see below). The right cremaster muscle was exposed through a ventral incision of the scrotum. The muscle was opened ventrally in a relatively avascular zone, using careful electrocautery to stop any bleeding, and spread over the transparent pedestal of a custom-made microscopy stage. Epididymis and testicle were detached from the cremaster muscle and placed into the abdominal cavity. Throughout the procedure as well as after surgical preparation during *in vivo* microscopy, the muscle was superfused with warm-buffered saline.

### *In vivo* microscopy

The setup for *in vivo* microscopy was centered around an Olympus BX 50 upright microscope (Olympus Microscopy, Hamburg, Germany), equipped for stroboscopic fluorescence epi-illumination microscopy. Light from a 75-W xenon source was narrowed to a near-monochromatic beam of a wavelength of 700 nm by a galvanometric scanner (Polychrome II, TILL Photonics, Graefelfing, Germany) and directed onto the specimen via a FITC filter cube equipped with dichroic and emission filters (DCLP 500, LP515, Olympus). Microscopic images were obtained with Olympus water immersion lenses [20x/numerical aperture (NA) 0.5 and 10x/NA 0.3] and recorded with an analog black-and-white charge-coupled device (CCD) video camera (Cohu 4920, Cohu, San Diego, CA, USA) and an analog video recorder (AG-7350-E, Panasonic, Tokyo, Japan). Reflected light oblique transillumination (RLOT) was obtained by positioning a mirroring surface (reflector) directly below the specimen and tilting its angle relative to the horizontal plane. The reflector consisted of a round cover glass (thickness, 0.19–0.22 mm; diameter, 11.8 mm), which was coated with aluminum vapor (Freichel, Kaufbeuren, Germany) and brought into direct contact with the overlying specimen as described previously [34]. For measurement of centerline blood flow velocity, green fluorescent microspheres (0.96 µm diameter, Molecular Probes, Leiden, The Netherlands) were injected via the femoral artery catheter, and their passage through the vessels of interest was recorded using the FITC filter cube under appropriate stroboscopic illumination (exposure, 1 ms; cycle time, 10 ms; λ = 488 nm), integrating video images for sufficient time (>80 ms) to allow for the recording of several images of the same bead on one frame. Beads that were flowing freely along the centerline of the vessels were used to determine blood flow velocity (see below).

### Quantification of leukocyte kinetics and microhemodynamic parameters

For off-line analysis of parameters describing the sequential steps of leukocyte extravasation, we used the Cap-Image image analysis software (Dr. Zeintl, Heidelberg, Germany). Rolling leukocytes were defined as those moving slower than the associated blood flow and quantified for 30 s. Firmly adherent cells were determined as those resting in the associated blood flow for more than 30 s and related to the luminal surface per 100 µm vessel length. Transmigrated cells were counted in regions of interest (ROI), covering 75 µm on both sides of a vessel over 100 µm vessel length. By measuring the distance between several images of one fluorescent bead under stroboscopic illumination, centerline blood flow velocity was determined. From measured vessel diameters and centerline blood flow velocity, apparent wall shear stress was calculated, assuming a parabolic flow velocity profile over the vessel cross section.

### Experimental groups

Animals were assigned randomly to the following groups: Sham-operated mice, mast cell-depleted mice as well as mice treated with tranexamic acid, ε-aminocaproic acid, aprotinin, cromolyn, MK-886, BN 52021, or drug vehicle undergoing I/R (30/120 min; n = 5–6). In another set of experiments, control mice received vehicle or different doses of recombinant murine plasmin administrated either by intraarterial (0.2, 2.0, and 20.0 µg; diluted in 200 µl PBS supplemented with 0.01% BSA) or by intrascrotal injection (0.02, 0.2, 2.0 µg; diluted in 200 µl PBS supplemented with 0.01% BSA; n = 3 each group). Additional experiments were performed in control mice, mast cell-depleted mice as well as in mice receiving either cromolyn, BN 52021, MK-886, or respective drug vehicle undergoing intrascrotal stimulation with plasmin (2.0 µg intrascrotally; n = 6 each group).

### Inhibitors and blocking antibodies

The following inhibitors were used: aprotinin (100.000 KIU kg^−1^ i.a.; 5 min prior to onset of reperfusion as a bolus and then as continuous infusion 100.000 KIU kg^−1^ h^−1^; Sigma-Aldrich, Deisenhofen, Germany) is a plasmin inhibitor [20]. BN 52021 (20 mg kg^−1^ i.a.; Sigma-Aldrich) is a PAF receptor antagonist; cromolyn (0.2 mg kg^−1^ i.a.; Sigma-Aldrich) is an inhibitor of mast cell degranulation [35]; ε-aminocaproic acid (100 mg kg^−1^ i.a. 5 min prior to onset of reperfusion as a bolus and then as continuous infusion 100 mg kg^−1^ h^−1^; Sigma-Aldrich) is a plasmin inhibitor [36]; MK-886 (1 mg kg^−1^ i.a.; Sigma-Aldrich) is an inhibitor of the 5-lipoxygenase activating protein (FLAP) hence blocking leukotriene synthesis [37]; tranexamic acid (100 mg kg^−1^ i.a. 5 min prior to onset of reperfusion as a bolus and then as continuous infusion 100 mg kg^−1^ h^−1^; Sigma-Aldrich) is a plasmin inhibitor [38]. Control animals received equivalent volumes of corresponding drug vehicles.

### Mast cell depletion

Mast cell depletion was performed as described elsewhere [39], [40]. Briefly, wild-type animals were treated with CMP 48/80 (1 mg/kg i.p.; Sigma-Aldrich, Deisenhofen, Germany) 48 h prior to experimentation. This approach has been observed previously to deplete mast cell granules and to allow time for CMP48/80-induced inflammation to dissipate.

### Experimental protocols

In a first set of experiments, three postcapillary vessel segments in a central area of the spread-out cremaster muscle were randomly chosen among those that were at least 150 µm away from neighboring postcapillary venules and did not branch over a distance of at least 150 µm. After having obtained baseline recordings of leukocyte rolling, firm adhesion, and transmigration in all three vessel segments, ischemia was induced by clamping all supplying vessels at the basis of the cremaster muscle using a vascular clamp (Martin, Tuttlingen, Germany). Stagnancy of blood flow was then verified by *in vivo* microscopy. After 30 min of ischemia, the vascular clamp was removed and reperfusion was restored for 140 min. Measurements, which took about 5 min, respectively, were repeated at 60 and 120 min after onset of reperfusion.

In a second set of experiments, leukocyte recruitment to the cremaster muscle was analyzed either 240 min after intraarterial (0.2, 2.0, or 20.0 µg) or 240 min after intrascrotal injection of plasmin (0.02, 0.2, or 2.0 µg in 0.2 ml PBS, Molecular Innovations, Novi, MI, USA). Five vessel segments were randomly chosen in a central area of the spread-out cremaster muscle among those that were at least 150 µm away from neighboring postcapillary venules and did not branch over a distance of at least 150 µm. After having obtained recordings of migration parameters, blood flow velocity was determined as described above. After *in vivo* microscopy, tissue samples of the cremaster muscle were taken for immunohistochemistry. Blood samples were collected by cardiac puncture for the determination of systemic leukocyte counts using a Coulter ACT Counter (Coulter Corp., Miami, FL, USA). Anesthetized animals were then killed by bleeding to death.

### Bone marrow-derived mast cells

BMMCs were derived from 8–12 week old C57BL/6J mice. Cells from fresh bone marrow were resuspended in complete Iscove's Modified Dulbecco's Medium (IMDM with 10% heat inactivated fetal calf serum [HIFCS], 2 mM L-Glutamine [Gln], 1% Penicillin-Streptomycin solution [PEST], 50 µM β-mercaptoethanol [β-ME], and 2 ng/ml recombinant murine IL-3 from Peprotech, Rocky Hill, NJ) and cultured at 37°C, 5% CO_2_ for four days. Subsequently, BMMCs were diluted weekly to 0.5×10^6^ cells/ml with a mixture of 80% fresh, complete IMDM and 20% recycled medium, with IL-3 added every 2^nd^ day. Nonadherent cells were monitored for the presence of FcεRI [with a phycoerythrin (PE)-conjugated hamster, anti-mouse FcεRIα antibody; clone MAR-1, eBioscience, San Diego, CA) and c-kit (rat IgG_2B_ anti-mouse CD117/c-kit; clone 3c1, ImmunoKontact, Bioggo, Switzerland) by FACS analysis.

### Microvascular permeability

In separate experiments, leakage of the macromolecule FITC dextran (5 mg in 0.1 ml saline, i.a., 5 min prior to induction of reperfusion; *M*r 150,000, Sigma-Aldrich) was analyzed in sham-operated control mice as well as in mice receiving tranexamic acid, ε-aminocaproic acid, aprotinin, cromolyn, or drug vehicle undergoing I/R (30/60 min). Five postcapillary vessel segments as well as the surrounding perivascular tissue were excited at 488 nm, and emission >515 nm was recorded by a CCD camera (Sensicam, PCO, Kelheim, Germany) within the 1^st^ minute of reperfusion as well as 10, 20, 30, 40, 50, and 60 min after induction of reperfusion using an appropriate emission filter (LP 515). Mean gray values of fluorescence intensity were measured by digital image analysis (TILLvisION 4.0, TILL Photonics) in six randomly selected ROIs (50x50 µm^2^), localized 50 µm distant from the postcapillary venule under investigation.

### Confocal microscopy

For the analysis of collagen IV expression, cremaster muscles were fixed in 4% paraformaldehyde. Tissues were then blocked and permeabilized in PBS, supplemented with 10% goat serum (Sigma-Aldrich) and 0.5% Triton X-100 (Sigma-Aldrich). After incubation with the primary rabbit anti-mouse collagen IV polyclonal antibody (Abcam, Cambridge, UK) at room temperature for 12 h, tissues were incubated with the secondary Alexa Fluor 488-linked goat anti-rabbit (Invitrogen, Carlsbad, CA, USA) antibody for 3 h at room temperature.

Immunostained tissues were mounted in PermaFluor (Beckman Coulter, Fullerton, CA, USA) on glass slides and observed using a Leica SP5 confocal laser-scanning microscope (Leica Microsystems, Wetzlar, Germany) with an oil immersion lens (Leica; 40x/NA 1.25–0.75). Optical sections of tissue samples through the whole depth of the tissue were obtained using, as far as possible, the same settings for all samples analyzed. Z-stack digital images were collected optically at every 0.5 µm depth and applied to three-dimensional (3D) reconstruction analysis using Leica Application Suite software. To analyze the expression profile of collagen IV, 3D images of vessels were split in the middle along the longitudinal axis. Images of these “semi-vessels” were then analyzed for fluorescence intensity, as described previously [16], [18], using Leica Application Suite software. Briefly, ROIs within 3D images of semi-vessels were identified manually, and their intensity profile was compared with the average intensity of the entire vessel within the same field of view. Collagen IV low-expression regions (LER) were defined as those regions in which the average fluorescence intensity/unit area was less than 60% of the average fluorescence intensity in the whole vessel segment under investigation. LE sites from three vessel segments/tissue (n = 4 mice per group) were analyzed. LE site size was determined using Leica Application Suite software, and LE site density was calculated for the total surface area of the semi-vessels.

Confocal microscopy on BMMCs was done after incubating the cells with TO-PRO 3 (Invitrogen, Carlsbad, USA) and a primary rabbit mAb directed against murine *histone protein H2B* (Abcam, Cambridge, UK) for 30 min, followed by incubation with secondary goat FITC-labeled anti-rabbit polyclonal antibody (Abcam). Image acquisition was performed as described above. Confocal planes covering the whole cell (projection of twenty five *z*-planes; *z*-spacing, 1 µm) were projected, using the maximum-intensity-projection module of Huygens software (SVI, Hilversum, Netherlands).

### Histochemistry

To determine mast cell activation *in vivo*, ruthenium red staining of the cremaster muscle was performed as described previously [41]. Mast cell activation was assessed in sham-operated control mice as well as in mice receiving tranexamic acid, ε-aminocaproic acid, aprotinin, cromolyn, MK-886, BN 52021, or drug vehicle undergoing I/R (30/120 min). Mast cell activation was also analyzed in control mice with an intrascrotal injection of PBS supplemented with 0.01% BSA as well as in mice receiving either cromolyn, MK-886, BN 52021, or drug vehicle undergoing intrascrotal stimulation with plasmin. As a positive control for mast cell staining, exteriorized cremaster muscles of untreated mice were superfused for 30 min with the mast cell activator compound 48/80 (CMP 48/80; 1 µg ml^-1^; Sigma Aldrich; n = 4 each group). Thereafter, exteriorized cremaster muscles were superfused for 60 min with a 0.001% solution of ruthenium red (Sigma Aldrich), respectively. The number of ruthenium red-positive cells was quantified by light microscopy (objective magnification 10x) in cremaster muscle whole mounts (10 observation fields per whole mount) from four individual animals per experimental group in a blinded manner, respectively.

### Immunohistochemistry

To determine the phenotype of transmigrated leukocytes, immunostaining of paraffin-embedded serial tissue sections of the cremaster muscle was performed. Sections were incubated with primary rat anti-mouse anti-Ly-6G, anti-CD45 (BD Biosciences, San Jose, CA, USA), or anti-F4/80 (Serotec, Oxford, UK) IgG antibodies. Then, the paraffin sections were stained with commercially available immunohistochemistry kits (Ly-6G, CD45, Super Sensitive Link-Label IHC detection system, BioGenex, San Ramon, CA, USA; F4/80, Vectastain ABC kit, Vector Laboratories, Burlingame, CA, USA), obtaining an easily detectable reddish or brownish end product, respectively. Finally, the sections were counterstained with Mayer's hemalaun. The number of extravascularly localized Ly-6G-, CD45-, or F4/80-positive cells was quantified by light microscopy (objective magnification 40x) on three sections (10 observation fields per section) from six individual animals per experimental group in a blinded manner, respectively. The number of transmigrated Ly-6G-positive cells (neutrophils/monocytes) and F4/80-positive cells (monocytes/macrophages) is expressed as the percentage of total CD45-positive leukocytes.

### Flow cytometry

BMMCs were incubated with primary rabbit mAb directed against murine *histone protein H2B* (Abcam, Cambridge, UK) for 30 min on ice. After washing, cells were incubated for 30 min on ice with a secondary goat FITC-labeled anti-rabbit polyclonal antibody (Abcam). Anticoagulated whole blood samples were incubated for 30 min at room temperature with different concentrations (1 and 100 ng ml^−1^) of plasmin or PMA (10 ng ml^−1^) as a positive control and with PBS supplemented with 0.01% BSA used as vehicle control. After washing, samples were incubated with fluorescence-labeled monoclonal antibodies (mAb) directed against either CD11b/Mac-1 (FITC) or CD62L/L-selectin (PE; BD Biosciences, San Jose, CA) on ice. Stained cells were analyzed on a flow cytometer (FACSort, Becton Dickinson, Franklin Lakes, NJ, USA). Approximately 10,000 gated events were collected in each analysis.

### RT-PCR

5-lipoxygenase (5-LO) and lyso-PAF-acetyltransferase (LPCAT) mRNA expression: Total RNA contents were extracted using the RNeasy Mini Kit (Qiagen GmbH, Hilden, Germany) according to the manufacturers' instruction manual. Reverse transcription was carried out using the High Capacity cDNA Reverse Transcription Kit (Applied Biosystems, Hamburg, Germany). Real time PCR was conducted with the TaqMan® Universal Master Mix Kit in a 7300 Real-Time PCR System (all Applied Biosystems, Hamburg, Germany). Primers and probes were obtained from ABI (Foster City, California, USA) TaqMan Gene Expression Assay catalog (5-LO: Mm01182750_m1; LPCAT: Mm00557141_m1) and from Biomers (Ulm, Germany) GAPDH forward: 5′-tgc agt ggc aaa gtg gag at-3′. GAPDH reverse: 5′- tgc cgt gag tgg agt cat act-3′ (bp 1-1254; gene bank accession number NM_008084); GAPDH TaqMan probe: 5′- FAM –cca tca acg acc cct tca ttg acc tc- BHQ-3′. GAPDH was used as an internal housekeeping gene. Calculation of the mRNA content was performed by a mathematical model developed by Pfaffl and collegues [42].

### Statistics

Data analysis was performed with a statistical software package (SigmaStat for Windows, Jandel Scientific, Erkrath, Germany). Unless otherwise stated, the ANOVA on ranks test followed by the Student-Newman-Keuls test was used for the estimation of stochastic probability in intergroup comparisons. Mean values and SEM are given. *P* values 0.05 were considered significant.
